# A comprehensive assessment using multiple factors based on HAS-Flow analysis predicts ATL development and progression

**DOI:** 10.1038/s41598-025-10822-4

**Published:** 2025-07-22

**Authors:** Hideaki Nakamura, Tatsuro Watanabe, Akemi Sato, Atsushi Kawaguchi, Kaoru Uchimaru, Yorifumi Satou, Eisaburo Sueoka

**Affiliations:** 1 Department of Transfusion Medicine, Saga University Hospital, Nabeshima 5-1-1, Saga, 849-8501 Japan; 2 Department of Drug Discovery and Biomedical Sciences, Faculty of Medicine, Saga University, Saga, Japan; 3 Department of Clinical Laboratory Medicine, Faculty of Medicine, Saga University, Saga, Japan; 4 Education and Research Center for Community Medicine, Faculty of Medicine, Saga University, Saga, Japan; 5 Laboratory of Tumor Cell Biology, Department of Computational Biology and Medical Sciences, Graduate School of Frontier Sciences, The University of Tokyo, Tokyo, Japan; 6 Division of Genomics and Transcriptomics, Joint Research Center for Human Retrovirus Infection, Kumamoto University, Kumamoto, Japan; 7 General Incorporated Association Yuai Social Welfare Organization, Next-Generation Medical Research Institute, Kashima, Saga, Japan

**Keywords:** HAS-Flow, ATL, HTLV-1, Prediction of development and progression of ATL, Identification of the HTLV-1 integration site, Tumour virus infections, Haematological cancer

## Abstract

Adult T-cell leukemia/lymphoma develops decades after Human T-lymphotropic virus type 1 (HTLV-1) infection. Factors like proviral load (PVL), soluble interleukin-2 receptors (sIL-2R), and clonality are associated with its pathogenesis. However, a comprehensive assessment using multiple factors of ATL development and progression based on flow cytometry (HAS-Flow) has not been performed. We conducted a 10-year clinical follow-up of 160 asymptomatic people living with HTLV-1 using HAS-Flow, PVL, sIL-2R, and the HTLV-1 integration site identification. The cases were classified into three groups based on cell adhesion molecule 1 (CADM1)-expressing cells by HAS-Flow: Group 1 (≤ 10%, 115 cases), Group 2 (> 10% to ≤ 25%, 33 cases), and Group 3 (> 25%, 12 cases). In the follow-up, no cases in Group 1 developed ATL, while five cases in Group 2 and nine in Group 3 did. Among the developed ATL, one case in Group 2 and six in Group 3 progressed to aggressive ATL. Higher CADM1-expressing cells and sIL-2R levels were linked to earlier ATL development. The HTLV-1 integration site was identified in all aggressive ATL cases. Thus, evaluating CADM1-expressing cells by HAS-Flow, assessing sIL-2R, and identifying the HTLV-1 integration site can better predict ATL development and progression to aggressive ATL.

## Introduction

Human T-lymphotropic virus type 1 (HTLV-1) primarily infects CD4-positive T cells, and the latest report estimates that at least 5–10 million people worldwide live with HTLV-1^1^. Although mother-to-child transmission of HTLV-1, the main route of HTLV-1 infection, has been effectively reduced through countermeasures in Japan^2^, in recent years, the horizontal transmission of HTLV-1 has been reported to be on the rise in Japan^3,4^. HTLV-1 is still an infectious disease that requires immediate action worldwide. The World Health Organization (WHO) has published various reports raising awareness of HTLV-1^5^. HTLV-1 infection causes many different types of diseases decades after infection, and the most widely known, with a poor prognosis, is adult T-cell leukemia/lymphoma (ATL)^6–8^. Diagnosis of ATL is based on histopathology of the tumor lesion or cytology, which shows more than 5% abnormal lymphocytes in the peripheral blood and positive serology for HTLV-1. According to the Shimoyama classification, ATL is classified into four subtypes (acute, lymphoma, chronic, and smoldering)^9^. Chronic ATL is divided into unfavorable or favorable according to the presence of any unfavorable prognostic factors, a high level of blood urea nitrogen (BUN), lactate dehydrogenase (LDH), or a low albumin level^10^. Despite the development of various therapeutic strategies, ATL prognosis remains poor^10–12^. Notably, in Japan, the lifetime risk of developing ATL in people living with HTLV-1 (same meaning as HTLV-1 carrier) is estimated to be approximately 5% (although this might be underestimated because of unreported cases), and most of them live as asymptomatic HTLV-1 carriers (ACs). Useful ways to distinguish ATL development are desired; however, there is no certainty yet.

Flow cytometry analysis has recently been developed to identify HTLV-1-infected cells using factors identified through comparative expression analysis of ATL patients and ACs. Several studies have reported that the flow cytometric analysis of CADM1 and CD7 in CD4-positive ATL cells is effective in predicting the progression and development of ATL^13–15^. The HTLV-1 analysis system was named HAS-Flow. A previous study reported older age, male, HTLV-1 proviral load (PVL), and family history of ATL risk factors for developing ATL^16^. In particular, PVL is a promising factor, and values of more than 4 copies per 100 peripheral blood mononuclear cells (PBMCs) have been reported to be associated with ATL development. Moreover, clonality and levels of soluble interleukin-2 receptors (sIL-2R) have been reported as factors related to the definitive diagnosis and prognosis of the aggressive type ATL (acute, lymphoma, and unfavorable chronic)^11,17,18^. Although each factor related to ATL development and progression has been reported, the relationships between HAS-Flow analysis and PVL, sIL-2R, and clonality in asymptomatic people living with HTLV-1 remain unclear. Furthermore, no long-term HAS-Flow analysis of more than 100 asymptomatic people living with HTLV-1 has been performed.

In this study, we analyzed 160 asymptomatic people living with HTLV-1 for up to 10 years to investigate whether HAS-Flow analysis and other factors can more accurately predict the development and progression of ATL.

## Results

### Categorization and clinical characteristics of asymptomatic people living with HTLV-1 by flow cytometric analysis, HAS-Flow

**Fig. 1:**
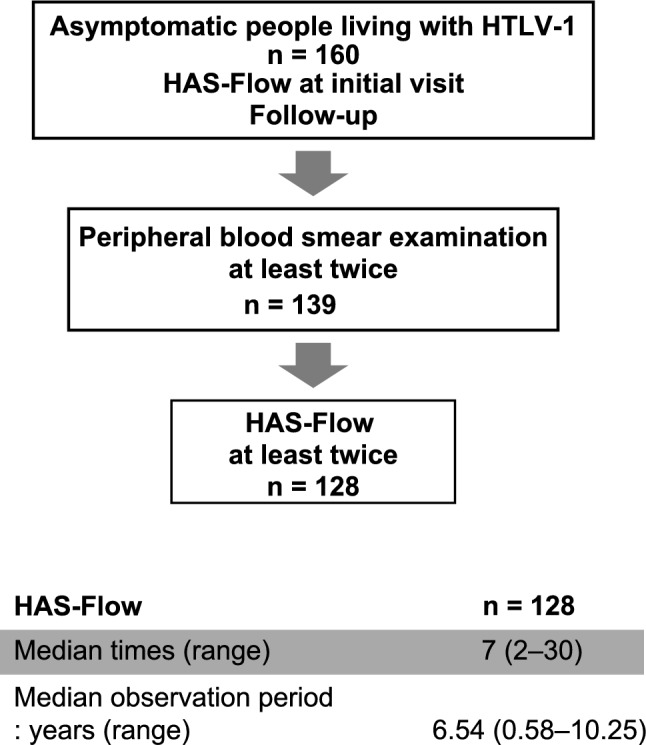
Flowchart of the cases analyzed in this study and details of the flow cytometry analysis (HAS-Flow) We performed HAS-Flow analysis on 160 asymptomatic people living with HTLV-1 on their first visit. During clinical follow-up after the initial examination, 139 cases underwent peripheral blood smear examinations at least twice. Furthermore, 128 of the 139 cases underwent HAS-Flow analysis at least twice during the clinical follow-up. Details of the number of analyses and the observation period of the HAS-Flow analysis are shown.

**Fig. 2:**
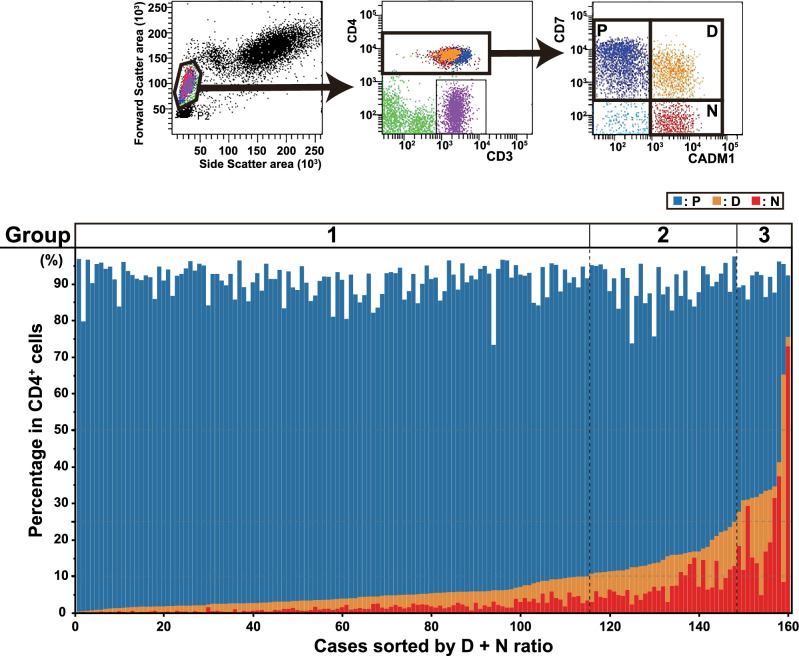
Classification of people living with HTLV-1 based on HAS-Flow analysis. The upper panel indicates the details of HAS-Flow analysis. Lymphocytes were first gated, and CD4-positive lymphocytes were categorized based on CD7 and CADM1 expression. CD7-positive and CADM1-negative cells were designated as P, CD7-positive and CADM1-positive cells, and CD7-negative and CADM1-positive cells were designated as D and N, respectively. The lower panel shows the results of the HAS-Flow analysis of 160 cases. The percentages of P (blue), D (orange), and N (red) in each case are shown in stacked bar graphs. The sum of D and N percentages in CD4-positive cells is shown from lower to higher, and the results are categorized into three groups (Group 1, D + N ≤ 10%; Group 2, 10% < D + N ≤ 25%; and Group 3, D + N > 25%). The horizontal dotted lines indicate 10 and 25% for classification. Vertical dotted lines represent grouping.

Between 2014 and 2024, 160 asymptomatic people living with HTLV-1 were enrolled in this study. Of them, 139 cases underwent peripheral blood-smear tests at least twice during follow-up after their first visit. Flow cytometric analysis to evaluate HTLV-1-infected cells, called HAS-Flow, was performed on 160 cases at the initial visit, and 128 cases were analyzed at least twice during the follow-up period. The details are shown in Fig. 1. The upper panel of Fig. 2 shows the details of the HAS-Flow analysis. First, lymphocytes were selected based on Forward Scatter and Side Scatter properties, and subsequently, CD4-positive lymphocytes were isolated. These were further classified based on CD7 and Cell Adhesion Molecule 1 (CADM1) expression. CD7-positive and CADM1-negative cells were identified as P, while CD7-positive and CADM1-positive cells and CD7-negative and CADM1-positive cells were identified as D and N, respectively. Previous studies indicate that CADM1-positive cells are prevalent in aggressive ATL^13,14^. Thus, we assessed the combined percentage of D and N cells expressing CADM1. Results were displayed as stacked bar graphs, arranged by ascending D + N percentage of HAS-Flow analysis, and divided into three categories: Group 1 (D + N ≤ 10%), Group 2 (10% < D + N ≤ 25%), and Group 3 (D + N > 25%), as depicted in Fig. 2, lower panel. Of the 160 cases, 115 (71.9%) were categorized as Group 1 (G1), 33 (20.6%) as Group 2 (G2), and 12 (7.5%) as Group 3 (G3). The categorized cases are summarized in Table 1. Age, sIL-2R, PVL, and percentage of abnormal lymphocytes were significantly different among the three groups. In contrast, no differences in age groups divided by age (40 years), sex, and white blood cell counts were observed. PVL measurements were performed in 31 cases in G2, eight cases in G3, and 24 and eight cases exceeded 4%, respectively.

**Table 1:** Details of the three groups classified by HAS-Flow.

	G1 (n = 115)	G2 (n = 33)	G3 (n = 12)	p-value
< 40 years old (%)	34 (29.6)	6 (18.2)	1 ( 8.3)	0.193^#^
≥ 40 years old (%)	81 (70.4)	27 (81.8)	11 (91.7)	
Female (%)	84 (73.0)	25 (75.8)	8 (66.7)	0.831^#^
Male (%)	31 (27.0)	8 (24.2)	4 (33.3)	
Age, median [range]	50 [24–86]	57 [28–82]	65 [39–83]	**0.021**^$^
sIL-2R (U/mL), median [range]	337 [128–972]	375 [206–1344]	602 [273–1761]	** < 0.001**^$^
PVL (%), median [range]	0.68 [0–7.6]	5.7 [1.8–16.7]	12.7 [4.1–63.7]	** < 0.001**^$^
Abnormal lymphocytes (%), median [range]	0 [0–4.5]	0.5 [0–4.5]	1.5 [0–4.5]	** < 0.001**^$^
WBC (X10^3^/uL), median [range]	6.3 [2.5–20.5]	5.9 [3.4–9.1]	6.3 [5.4–10.4]	0.211^$^

sIL-2R, soluble interleukin-2 receptors; PVL, HTLV-1 proviral load; WBC, white blood cell counts.

^#^Fisher’s exact test, ^$^Kruskal–Wallis tests.

Significant values are in bold.

### Characteristics and clinical course of the 14 patients who developed ATL

Following the initial HAS-Flow analysis, 139 of the 160 cases (G1:96; G2:31; G3:12) were followed for approximately 10 years. During this period, no cases in G1 developed ATL, whereas ATL was diagnosed in five cases in G2 and nine cases in G3. Among those diagnosed with ATL, one case in G2 and six cases in G3 subsequently progressed to aggressive ATL. Detailed information regarding these ATL cases is presented in Table 2.

**Table 2:** Details of the 14 patients who developed ATL.

Pt No	Final clinical type	Initial data								Smoldering ATL					Aggressive ATL				
		Age	Sex	HAS-Flow:D + N (%)	HAS-Flow:Group	WBC (X10^3^/μL)	Ly (%)	Abnomal Ly (%)	Ly (X10^3^/μL)	WBC (X10^3^/μL)	Ly (%)	Abnomal Ly (%)	Ly (X10^3^/μL)	Duration (years)	WBC (X10^3^/μL)	Ly (%)	Abnomal Ly (%)	Ly (X10^3^/μL)	Duration (years)
A34	Smoldering	71	F	11.9	G2	4.9	38.5	0.5	1.9	4	32.5	5	1.5	5.2					
A47	Smoldering	44	F	11.5	G2	6	27.5	0	1.7	3.9	26.5	10.5	1.4	0.9					
A151	Smoldering	41	F	23.5	G2	8.8	30.5	3	2.9	7	27.5	6	2.3	1.7					
A114	Smoldering	56	F	16.6	G2	7.2	32	1	2.4	7.7	25.5	5.5	2.4	7.6					
A267	Lymphoma	69	M	16.8	G2	4.5	33	0	1.5						5.5	14	0	0.8	3.2
A4	Smoldering	56	F	27.7	G3	7.9	32.5	0	2.6	4.8	37	5	2.0	3.4					
A87	Smoldering	66	F	32.6	G3	6.2	32	3.5	2.2	6.1	34.5	7.5	2.6	0.3					
A326	Smoldering	67	F	33.7	G3	8.7	38	2	3.5	6.5	33	5.5	2.5	0.3					
A9	Unfavarable chronic *^1^	83	M	31	G3	5.5	29	4.5	1.8	7.1	24	5	2.1	1.7	9.4	1	45.5	4.4	2.9
A20	Unfavarable chronic *^2^	81	F	65.2	G3	10.4	23.5	1.5	2.6	11.8	10.5	7.5	2.1	0.2	8.1	48	10	4.7	1.7
A23	Acute	77	F	41.2	G3	6.1	38	3.5	2.5	6.7	22	9.5	2.1	2.0	4.6	9.5	5	0.7	1.1
A25	Acute	64	M	31.5	G3	5.9	34	1.5	2.1	6.2	35.5	7	2.6	1.3	9.2	23	31	5.0	4.9
A53	Unfavarable chronic *^1^	55	F	34.6	G3	7	18.5	1	1.4	8.3	29	9	3.2	1.6	9.3	11.5	35.5	4.4	0.5
A210	Unfavarable chronic *^3^	58	F	75.5	G3	6.2	41	0	2.5	8.2	21.5	7.5	2.4	0.2	7.7	55	7	4.8	3.0

WBC, white blood cell counts; Ly, lymphocyte.

*1, Blood urea nitrogen (BUN) and lactate dehydrogenase (LDH) excessed the upper limit of normal. *2, LDH excessed the upper limit of normal. *3, BUN excessed the upper limit of normal.

### Correlations of the D + N percentage of HAS-Flow analysis and other factors related to ATL development and progression

**Fig. 3:**
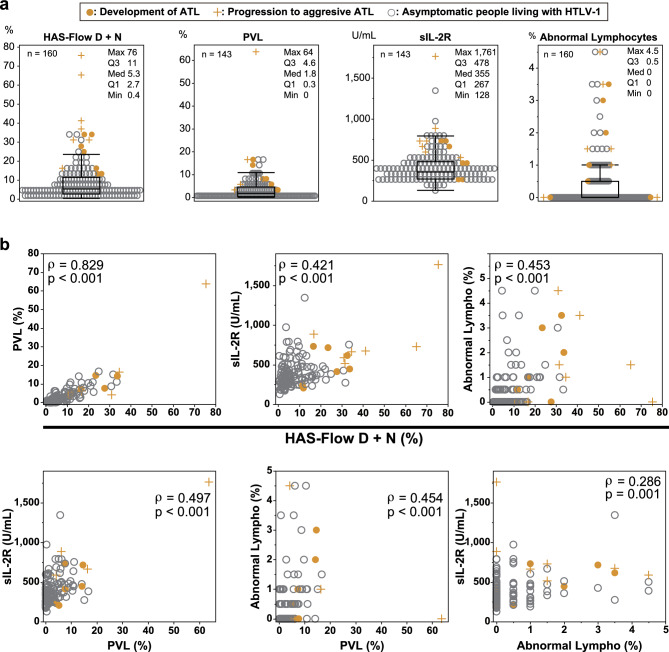
Correlation of D + N percentage of HAS-Flow analysis and other factors related to ATL development and progression (**a**) The data distribution of the examined markers at the first visit of people living with HTLV-1 (D + N percentage of HAS-Flow analysis, Proviral load: PVL, Soluble interleukin-2 receptors: sIL-2R, and percentage of abnormal lymphocytes) is shown as a scatter plot with a box plot. Analyzed numbers and box plot details are displayed in the top left or right, respectively. (**b**) Six scatter plots of combinations of the four values above are shown. Correlations were examined using Spearman rank correlation analysis, and the values are shown in the upper area. The orange dots represent cases that developed adult T-cell leukemia/lymphoma (ATL), and the orange plus marks represent cases that progressed to aggressive ATL. The gray circles represent cases of non-developed ATL.

We examined the relationship between the HAS-Flow analysis and factors related to ATL development and progression (PVL, sIL-2R, and percentage of abnormal lymphocytes) collected at the same time. Figure 3a shows the distribution of the examined results at the first visit in asymptomatic people living with HTLV-1. In Fig. 3, of the 14 cases that developed ATL during follow-up, seven that did not progress are indicated by orange dots, and 7 that progressed to aggressive ATL are indicated by orange plus marks. Almost all cases that developed ATL were located at or above the third quartile (Q3) of the D + N percentage in the HAS-Flow analysis. As in HAS-Flow, developed ATL cases tended to be observed in Q3 and above in PVL. Meanwhile, some cases of developed ATL were observed below Q3 in sIL-2R and the percentage of abnormal lymphocytes (Fig. 3a). We also performed a correlation analysis of all the data. A strong correlation was observed between the D + N percentage in HAS-Flow analysis and PVL (ρ = 0.829, *P* < 0.001). Additionally, a moderate correlation was observed between the D + N percentage and sIL-2R (ρ = 0.421, *P* < 0.001) and the percentage of abnormal lymphocytes (ρ = 0.453, *P* < 0.001) (Fig. 3b). In addition, a moderate correlation was observed between PVL, sIL-2R (ρ = 0.497, *P* < 0.001), and the percentage of abnormal lymphocytes (ρ = 0.454, *P* < 0.001). The correlation between sIL-2R and the percentage of abnormal lymphocytes was the lowest (ρ = 0.286, *P* = 0.001) (Fig. 3b).

### Relationships between the classification based on the D + N percentage of HAS-Flow analysis and ATL development or progression

**Fig. 4:**
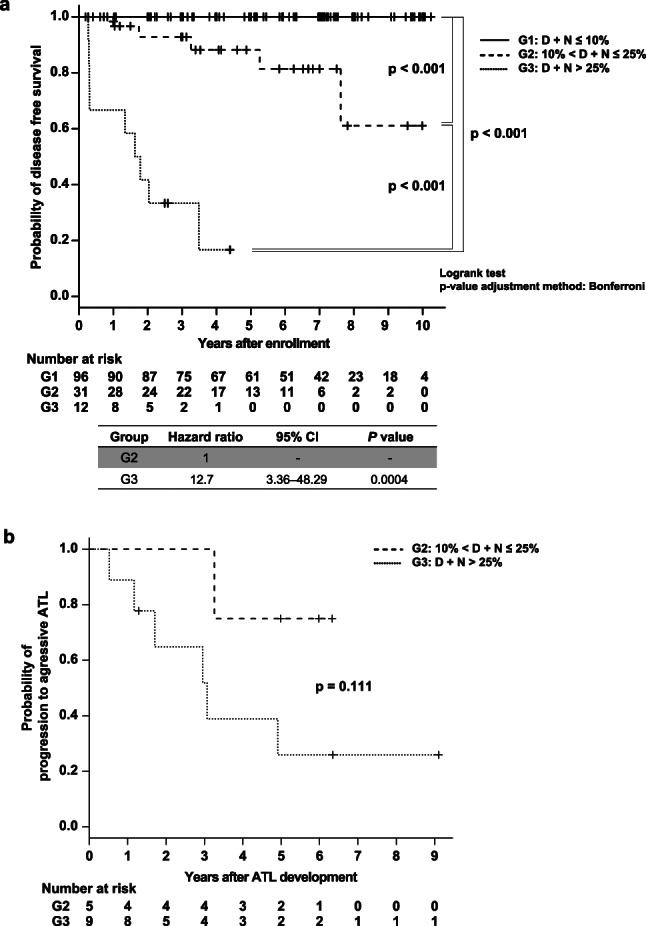
Probability of ATL disease-free or aggressive ATL-free survival for the three groups classified by the D + N percentage in HAS-Flow analysis Adult T-cell leukemia/lymphoma (ATL)-free and aggressive ATL-free survival analysis of the three groups classified based on HAS-Flow analysis was performed using the Kaplan–Meier method. (**a**) The number of years between the initial HAS-Flow analysis and the last peripheral blood smear was evaluated, and ATL development was defined as having ≥ 5% abnormal lymphocytes on examination. (**b**) Time from ATL development to aggressive ATL was assessed. The censored values (+) indicate the last known follow-up time of the participants who were alive without developing or aggressive ATL. A log-rank test with Bonferroni correction was performed, and the respective *p*-values are shown. The Cox proportional hazards model was used, and the hazard ratios and 95% confidence intervals are shown below the figure.

To clarify the association between the D + N percentage of HAS-Flow analysis and the development of ATL, we assessed the time to ATL onset using survival curve analysis (Fig. 4a). A log-rank test with Bonferroni correction showed statistical significance between G2 and G3 (*P* < 0.001) and between G2 (*P* < 0.001) and G3 (*P* < 0.001) compared with G1. In G3, the time to ATL development was particularly short. The hazard ratio calculated by the Cox proportional hazard model for G3 to G2 was 12.7 (95% CI: 3.36–48.29). Furthermore, among the development cases of ATL, one and six individuals in G2 and G3, respectively, progressed to aggressive ATL. We also evaluated the time to aggressive ATL from ATL development as in the above-mentioned analysis (Fig. 4b). Statistical significance was not observed between G2 and G3 (P = 0.111). In G3, the median time to progression to aggressive ATL after ATL development was around 3 years.

### Continuous analysis of HAS-Flow in asymptomatic people living with HTLV-1

**Fig. 5:**
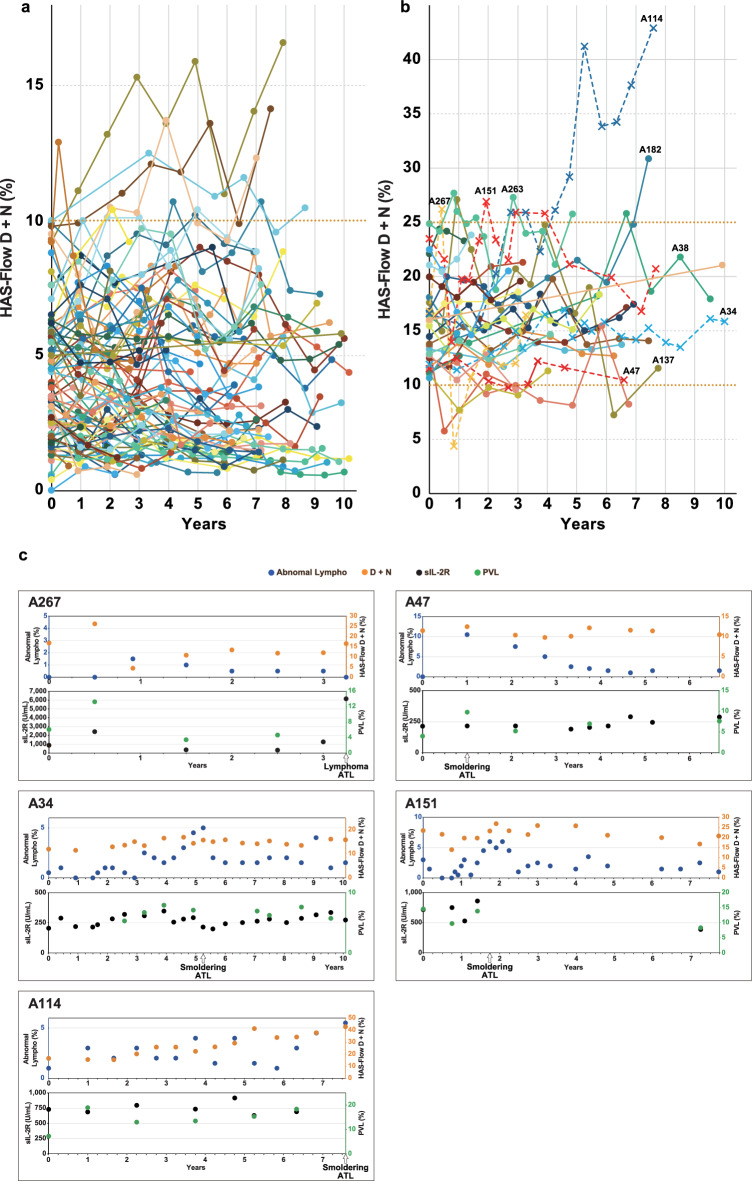

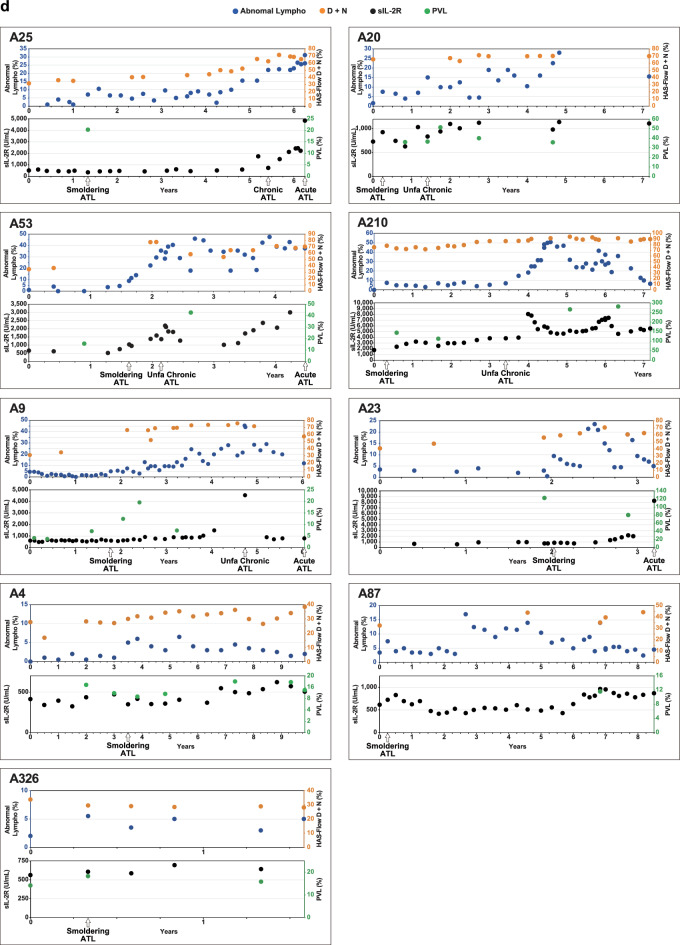
Continuous HAS-Flow analysis of people living with HTLV-1 and sequential changes of clinical markers in 14 cases that developed ATL A continuous HAS-Flow analysis was performed for up to 10 years, and the D + N percentage in each case is shown as a line graph. In this figure (**a**, **b**) show the data from Group 1 (G1) and Group 2 (G2) categorized by HAS-Flow analysis, respectively. The cross marks and dotted line in G2 indicate cases that developed adult T-cell leukemia/lymphoma (ATL) during follow-up, and those with significant changes in G2 are numbered. The horizontal orange dotted lines indicate 10% and 25% for classification. Changes in four clinical markers (percentage of abnormal lymphocytes, D + N percentage of HAS-Flow analysis, Soluble interleukin-2 receptors: sIL-2R, and Proviral load: PVL) in five cases that developed ATL in G2 are shown in (**c**). Similar to (**c**), nine cases that developed ATL in Group 3 are shown in (**d**). The timing of ATL development and progression is indicated by the arrows below. Unfavorable chronic ATL is showed as Unfa Chronic.

After the initial HAS-Flow analysis, 128 cases underwent HAS-Flow analysis two or more times. We assessed HAS-Flow analysis during long-term clinical follow-up. Serial changes in the D + N percentage of HAS-Flow analysis in G1 and G2 cases are shown in Fig. 5a, b. The longest and shortest observation periods were 10.3 years and 7 months, respectively. In G1, 87 cases were observed, and nine cases occasionally exceeded 10% of the D + N percentage (Fig. 5a). In G2, seven of 30 cases occasionally exceeded 25% of the D + N percentage (Fig. 5b). No development of ATL was observed in G1; however, five cases developed ATL in G2, as indicated by the cross mark and dotted line. The detailed data are shown in Fig. 5c. In case A267, the D + N percentage of HAS-Flow analysis exceeded 25% after the first analysis, and the sIL-2R exceeded 1000 U/mL. Approximately 3 years after enrollment, the case was diagnosed with aggressive ATL lymphoma. On the other hand, the other cases (A34, A47, A151, and A114) were diagnosed with smoldering ATL of low-grade. Furthermore, sIL-2R never exceeded 1000 U/mL in these cases. We also performed a longitudinal evaluation of G3. As mentioned above, six cases (A9, A20, A23, A25, A53, and A210) progressed to aggressive-type ATL in G3. Detailed clinical follow-up data of ATL development in G3 are shown in Fig. 5d. In cases that progressed to aggressive ATL, the D + N percentage of HAS-Flow analysis gradually increased with ATL progression. Furthermore, the sIL-2R levels exceeded 1000 U/mL before progression to aggressive ATL. On the other hand, the cases with smoldering ATL (A4, A87, and A326) showed no remarkable changes in the D + N percentage of HAS-Flow analysis and never had sIL-2R levels exceeding 1000 U/mL.

### Identification analysis of the HTLV-1 integration site in nine cases each from G2 and G3

**Fig. 6:**
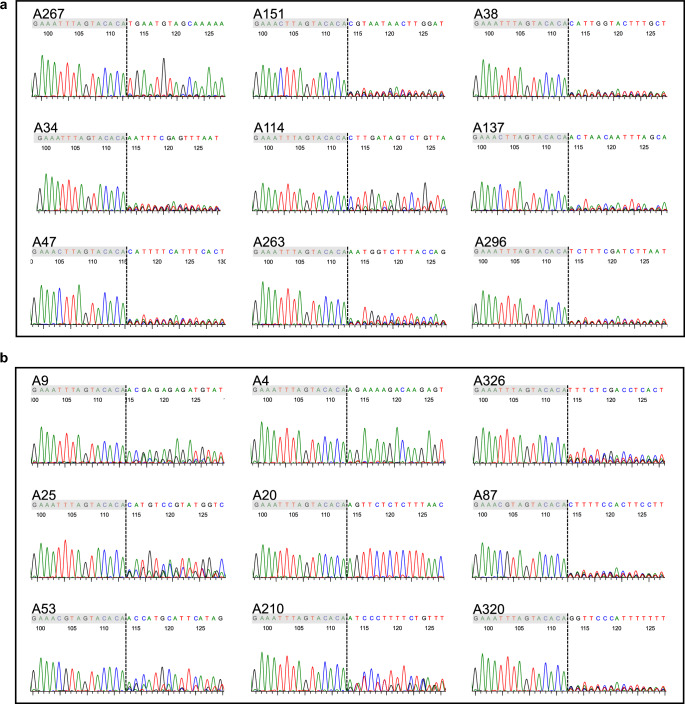
Analysis of the integration site of HTLV-1 into the host genome in nine cases each from Group 2 and Group 3, categorized by HAS-Flow analysis Integration site analysis using Rapid Amplification of Integration Sites without Interference by Genomic DNA contamination (RAISING) was performed, and the Sanger sequences are shown. The sequence with a gray background represents HTLV-1, and the next sequence and dotted line represent the host genome and integration site, respectively. In this figure (**a**) shows the nine cases from Group 2. Case A267 progressed to aggressive adult T-cell leukemia/lymphoma (ATL), and cases A34, A47, A151, and A114 developed smoldering ATL. In this figure (**b**) shows the nine cases from Group 3. Cases A9, A25, A53, A20, and A210 progressed to aggressive ATL, and cases A4 and A326 were smoldering ATL.

**Fig. 7:**
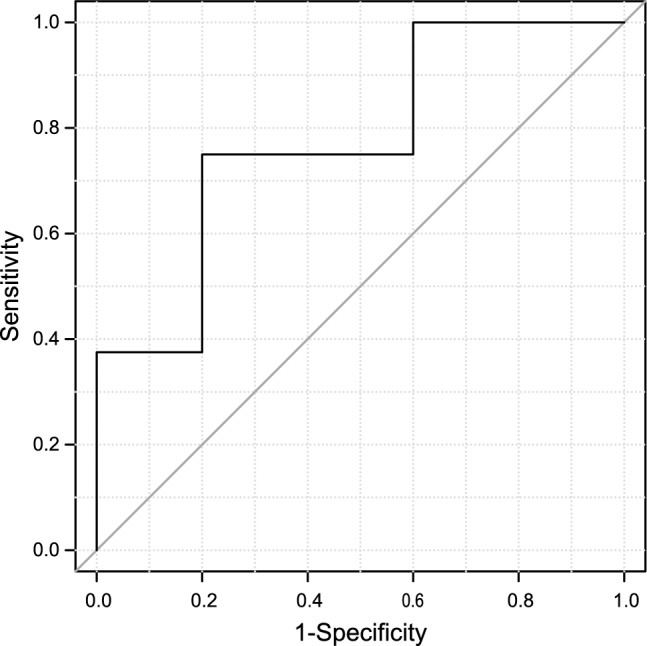
Receiver operating characteristic (ROC) curve analysis of HAS-Flow for detecting monoclonal population. The ROC curve was generated using 18 cases with integration analysis to evaluate the diagnostic performance of HAS-Flow. The true positive rate (sensitivity) is plotted against the false positive rate (1—specificity) at various threshold settings. The area under the curve (AUC) was 0.775 (95% CI 0.546–1.000), indicating a moderate discriminative ability. The gray line represents the performance of a random classifier (AUC = 0.5).

To assess a more accurate prognosis of ATL onsets, we evaluated the clonality of HTLV-1 using the method of Rapid Amplification of Integration Sites without Interference by Genomic DNA contamination (RAISING)^19^. HTLV-1 DNA, typically integrated as a single copy into the host cell’s genome, allows the determination of clonal expansions as monoclonal, oligoclonal, or polyclonal. Integration sites can be identified in monoclonal expansions indicative of aggressive ATL. We analyzed HTLV-1 clonality in nine cases each from G2 and G3. In G2, monoclonal integration sites were detected in three cases (A267, A114, and A263), while others were not (Fig. 6a). The integration site of HTLV-1 in G3 was identified in seven cases, but not in two cases (A87 and A320) (Fig. 6b). Of the seven cases, five cases (A9, A25, A53, A20, and A210) progressed to aggressive type ATL, and two cases (A4 and A326) were smoldering ATL. Patients who underwent RAISING analysis before and after progression to aggressive ATL had the same HTLV-1 integration site. Detailed integration site data are available in Supplementary Table S1. To assess the sensitivity and specificity of HAS-Flow in detecting a monoclonal population, we performed a receiver operating characteristic (ROC) analysis using 18 cases with integration analysis. The area under the ROC curve (AUC) was 0.775 (95% CI 0.546–1.000), indicating a moderate discriminative ability. The optimal cutoff value, determined by the Youden index, was 23.5, with a sensitivity of 0.75 and a specificity of 0.80 (Fig. 7).

## Discussion

A considerable proportion of people living with HTLV-1 remain asymptomatic throughout their lifetime. Nonetheless, psychological distress may arise among asymptomatic people living with HTLV-1 due to apprehensions regarding the potential development of ATL. To optimize the quality of life (QOL) in this population, it is imperative to stratify the risk of ATL development and evaluate long-term prognostic indicators. Accurate prediction of ATL development in asymptomatic HTLV-1-infected individuals could facilitate the implementation of targeted prophylactic interventions. Moreover, once ATL develops, many cases evolve into the aggressive subtype, which is associated with poor clinical outcomes. Consequently, early detection of ATL progression is essential for improving therapeutic efficacy and patient prognosis. The utility of flow cytometry in evaluating HTLV-1-infected cells has been reported. In the previous report, the result of HAS-Flow analysis was categorized into four groups (G1, D + N ≤ 10%; G2, 10% < D + N ≤ 25%; G3, 25% < D + N ≤ 50%; and G4, 50% < D + N)^15^. In G4, out of 14 cases, two were asymptomatic, while the remaining cases comprised six smoldering and six chronic ATL. This study aimed to predict ATL development in asymptomatic people living with HTLV-1, and only two cases showed more than 50% D + N percentage in HAS-Flow analysis. Therefore, cases displaying a D + N percentage of HAS-Flow analysis exceeding 50% were included in Group 3. We conducted HAS-Flow analysis on 160 asymptomatic people living with HTLV-1 at their initial visit and monitored their clinical follow-up for up to 10 years. No ATL development was noted when the D + N percentage of HAS-Flow analysis was under 10%. Conversely, among those with a percentage over 25%, nine out of 12 cases developed ATL during the follow-up. Moreover, six out of nine cases progressed to aggressive ATL. There were three cases (A206, A320, and A332) without ATL development in G3 during clinical follow-up. In A206, clinical follow-up was inadequate, and only one HAS-Flow analysis was performed. The follow-up period of A320 and A332 was 2.6 and 3.0 years, respectively. In addition, the A320 was 39 years old when it was registered. These cases could indicate that longer and more detailed clinical follow-up is needed to determine the ATL development. These findings suggest a low risk of ATL development when the initial HAS-Flow analysis D + N percentage is below 10%, and a high risk when it exceeds 25%. A previous report supported the findings^15^. In cases with D + N percentages greater than 10% and less than 25%, five out of 33 cases developed ATL in the follow-up period, and this category could have an intermediate risk for ATL development. Of the five cases that developed ATL, two (A34 and A47) had a D + N percentage of HAS-Flow analysis lower than 15% and never exceeded 20% during follow-up. The remaining three cases (A267, A151, and A114) had a D + N percentage greater than 15% at the initial analysis and over 25% during the follow-up, with A267 eventually progressing to lymphoma-type ATL. These cases showed variations in sIL-2R; A267, A151, and A114 had sIL-2R exceeding 500 U/mL, while A34 and A47 remained below this threshold (Fig. 5c). Elevated sIL-2R has been reported to correlate with the prognosis of ATL; however, they are also observed in other pathological conditions^20,21^. Combined with previous reports and our results, the combining the evaluation of HAS-Flow analysis with sIL-2R could more accurately predict the development of ATL or its prognosis.

Aggressive ATL is characterized by the monoclonal proliferation of HTLV-1-infected cells, which is diagnosed by the identification of HTLV-1 DNA integrated into a single site in the host DNA^17,22^. Therefore, we performed HTLV-1 integration site analysis and investigated the prognosis after ATL development by combining this approach with HAS-Flow analysis. All cases that progressed to aggressive ATL exhibited HTLV-1 integration sites. Furthermore, the case (A263) with an identified HTLV-1 integration site demonstrated a tendency toward an increased D + N percentage in HAS-Flow analysis during clinical follow-up (Supplementary Fig. S1). These findings suggest that the combination of HAS-Flow analysis, sIL-2R evaluation, and the identification of HTLV-1 integration sites is useful for predicting ATL development and progression. We propose the following as an example of an efficient workflow: first, HAS-Flow analysis is used to identify cases at intermediate and high risk of ATL development. In these cases, continuous HAS-Flow analysis and sIL-2R measurements should be performed during clinical follow-up, and HTLV-1 integration site identification analysis ought to be added depending on the clinical condition. A previous study reported that quantifying cell clonality in ATL via T-cell receptor subunit analysis using flow cytometry can detect early development of ATL^23^.

PVL is a known risk factor for the development of ATL^16^; however, its correlation with other factors is unknown. Our results showed a strong correlation between the D + N percentage of HAS-Flow analysis and PVL, and the distribution of the data in the box plots was similar (Fig. 3). A previous report described that the cut-off value for the development of ATL was 4% of PVL^16^, which is close to Q3 (4.7%) of the box plot of our data. A previous study reported that almost all cases with > 4% PVL exceeded 10% of the D + N percentage of HAS-Flow analysis^14^. Therefore, 10% of the D + N percentage of HAS-Flow analysis, which is located close to Q3 (11%) of the box plot, is a highly potentiated criterion for predicting the development of ATL. Additionally, the upper limit of the whisker of the box plot of the D + N percentage of HAS-Flow analysis is close to 25%. This indicates that values above 25% are considered outliers, and we consider this value to be significant for use as a threshold value. PVL and HAS-Flow analysis have unique characteristics. PVL levels were higher in ATL patients than in asymptomatic people living with HTLV-1^24^; moreover, small prospective studies have analyzed PVL levels in asymptomatic people living with HTLV-1^24,25^. Although PVL has been analyzed in more than 1000 asymptomatic people living with HTLV-1 to date^16^, the changes in PVL during long-term clinical follow-up and its relationship with ATL progression remain unclear. HAS-Flow analysis has been continuously reported^13–15,26^. Previous reports and this study have made it possible for HAS-Flow analysis to distinguish between high- and low-risk groups of ATL development in asymptomatic people living with HTLV-1. In addition, the D + N percentage of HAS-Flow analysis reflects the progression of ATL^13^, and a reduction in this percentage has been observed in the effective cases with chemotherapies for ATL^27,28^. These findings indicate that HAS-Flow analysis can provide meaningful information for the clinical follow-up of ATL.

This study has some limitations and future issues. First, the study was conducted at a single institution, and validation across multiple institutions may be necessary. Second, predicting the lymphoma-type of ATL using peripheral blood and HAS-Flow-based analyses proved challenging. The primary reason is that lymphoma type ATL presents with very few ATL cells in the peripheral blood. To resolve this, more sensitive markers and detection methods are necessary. A recently reported method that evaluates PVL using cell-free DNA and digital PCR has shown promise^29^. Third, we demonstrated the importance of sIL-2R, but further studies exploring the relationship between the development of ATL and sIL-2R are warranted. Finally, recent advances in whole-genome sequencing of ATL have elucidated the landscape of gene alterations in ATL^30–32^. Combining gene alteration analysis with HAS-Flow analysis could enable a more accurate prediction of the development and progression of ATL; however, further investigation is required.

In conclusion, this is the first study to perform HAS-Flow analysis on over 100 asymptomatic people living with HTLV-1 at their initial visit and to observe their long-term clinical follow-up. We demonstrated that the evaluation of HAS-Flow analysis is beneficial for predicting the development and progression of ATL. Additionally, the combination of HAS-Flow analysis with sIL-2R and identification of the integration site enhanced prediction accuracy. We believe that our findings will greatly contribute to the early diagnosis and treatment of ATL.

## Methods

### Samples and clinical data

All studies using human samples were performed following the guidelines of the Declaration of Helsinki and approved by the Institutional Review Board of Saga University (24–52 and 2018-03-02). Cases with positive HTLV-1 serology and a peripheral blood smear showing < 5% abnormal lymphocytes were enrolled. After enrollment, peripheral blood samples were collected, and complete blood counts were obtained from medical records. In this study, ATL was diagnosed in people living with HTLV-1 when more than 5% of abnormal lymphocytes were present in the peripheral blood, together with other clinical and pathological findings. Aggressive ATL is diagnosed in HTLV-1-positive individuals who show rapid disease progression, ≥ 5% abnormal lymphocytes in peripheral blood, elevated LDH levels, hypercalcemia, and involvement of multiple organs such as lymph nodes, liver, spleen, skin, or CNS. It includes the acute, lymphoma, and unfavorable chronic ATL subtypes. Serum levels of sIL-2R and PVL were obtained from the Joint Study on Predisposing Factors of ATL Development (JSPFAD). PVL analysis was performed according to previous reports^16,33^, and detailed primers used are listed in Supplementary Table S2. Briefly, the genomic DNA of normal control PBMCs mixed with a plasmid DNA containing the HTLV-1 provirus was used as the control template. To calculate the input cell number, RNase P was used as an internal control. In this study, 1 copy/100 PBMCs was defined as 1%.

### Flow cytometry

Analysis was performed according to a previous report^34^. Briefly, whole blood or PBMCs were stained using the following antibodies: CADM1-Alexa Fluor 647 (MBL), CD3-APC-H7, CD4-PerCP-Cy5.5, and CD7-PE (BD Biosciences, San Jose, CA, USA). After incubation, whole blood samples were lysed using a BD FACS Lyse Wash Assistant, and the isolated PBMCs were washed. After staining, the cells were analyzed using a FACSCanto II, and the results were analyzed using FlowJo version 10.4.1 software (BD, San Jose, CA, USA; https://www.flowjo.com/).

### Integration site analysis

We performed integration site analysis according to a previous report^19^. Briefly, single-strand DNA (ssDNA) synthesis of the isolated genomic DNA from PBMCs was performed, and the sample was purified. Poly-A was added to the purified ssDNA using TdT, and double-stranded DNA synthesis was performed. Moreover, double-stranded DNA was amplified using two rounds of PCR, and the fragment was purified using a QIAquick PCR Purification Kit. Direct sequence analysis of the fragment was performed using the BigDye Terminator Cycle Sequencing Kit (Applied Biosystems, Foster City, CA, USA) and SeqStudio according to the manufacturer’s instructions. The integration site of the host genome was analyzed using sequencing analysis software and the GGGenome web tool (https://gggenome.dbcls.jp/ja/). Detailed primers used are listed in Supplementary Table S3.

### Statistical analysis

Categorical and continuous data from different groups were compared using Fisher’s exact and Kruskal–Wallis tests, respectively. Correlation analysis of the markers was performed using Spearman’s rank correlation test. For the analysis of ATL-free survival and time to aggressive ATL progression from ATL development, the Bonferroni-corrected log-rank test and the Cox proportional hazards model were used. For the analysis of ATL-free survival and time to progression to aggressive ATL, the starting point was defined as either the first HAS-Flow analysis or ATL development, and the endpoint was defined as ATL development or progression to aggressive ATL. Statistical significance was set at *P* < 0.05. Statistical analyses were performed using JMP Pro software (version 17.0) and EZR software (Saitama Medical Centre, Jichi Medical University, Saitama, Japan).

## Supplementary Information

Supplementary Information.

## Data Availability

The Sanger sequencing data in this study can be found in the figshare. (10.6084/m9.figshare.28899731) The data supporting the findings of this study are available from the corresponding author upon reasonable request.

## Abbreviations

ACs: Asymptomatic HTLV-1 carriers
ATL: Adult T-cell leukemia/lymphoma
CADM1: Cell adhesion molecule 1
CI: Confidence interval
CD: Cluster of differentiation
HAS-Flow: HTLV-1 analysis system using flow cytometry
HTLV-1: Human T-lymphotropic virus type 1
JSPFAD: Joint study on predisposing factors for ATL development
PBMCs: Peripheral blood mononuclear cells
PVL: Proviral load
RAISING: Rapid amplification of integration sites without interference by genomic DNA contamination
sIL-2R: Soluble interleukin-2 receptors
TdT: Terminal deoxynucleotidyl transferase
WHO: World Health Organization
