# Correction: A comprehensive assessment using multiple factors based on HAS-Flow analysis predicts ATL development and progression

**DOI:** 10.1038/s41598-025-31394-3

**Published:** 2025-12-12

**Authors:** Hideaki Nakamura, Tatsuro Watanabe, Akemi Sato, Atsushi Kawaguchi, Kaoru Uchimaru, Yorifumi Satou, Eisaburo Sueoka

**Affiliations:** 1 Department of Transfusion Medicine, Saga University Hospital, Nabeshima 5-1-1, Saga, 849-8501 Japan; 2 Department of Drug Discovery and Biomedical Sciences, Faculty of Medicine, Saga University, Saga, Japan; 3 Department of Clinical Laboratory Medicine, Faculty of Medicine, Saga University, Saga, Japan; 4 Education and Research Center for Community Medicine, Faculty of Medicine, Saga University, Saga, Japan; 5 Laboratory of Tumor Cell Biology, Department of Computational Biology and Medical Sciences, Graduate School of Frontier Sciences, The University of Tokyo, Tokyo, Japan; 6 Division of Genomics and Transcriptomics, Joint Research Center for Human Retrovirus Infection, Kumamoto University, Kumamoto, Japan; 7 General Incorporated Association Yuai Social Welfare Organization, Next-Generation Medical Research Institute, Kashima, Saga, Japan

Correction to: *Scientific Reports* 10.1038/s41598-025-10822-4, published online 22 July 2025

The original version of this Article contained an error in Fig. 4, panel a, where the hazard ratio calculated by the Cox proportional hazard model for G3 to G2 was incorrect. The original Fig. 4 and accompanying legend appear below.

**Fig. 4:**
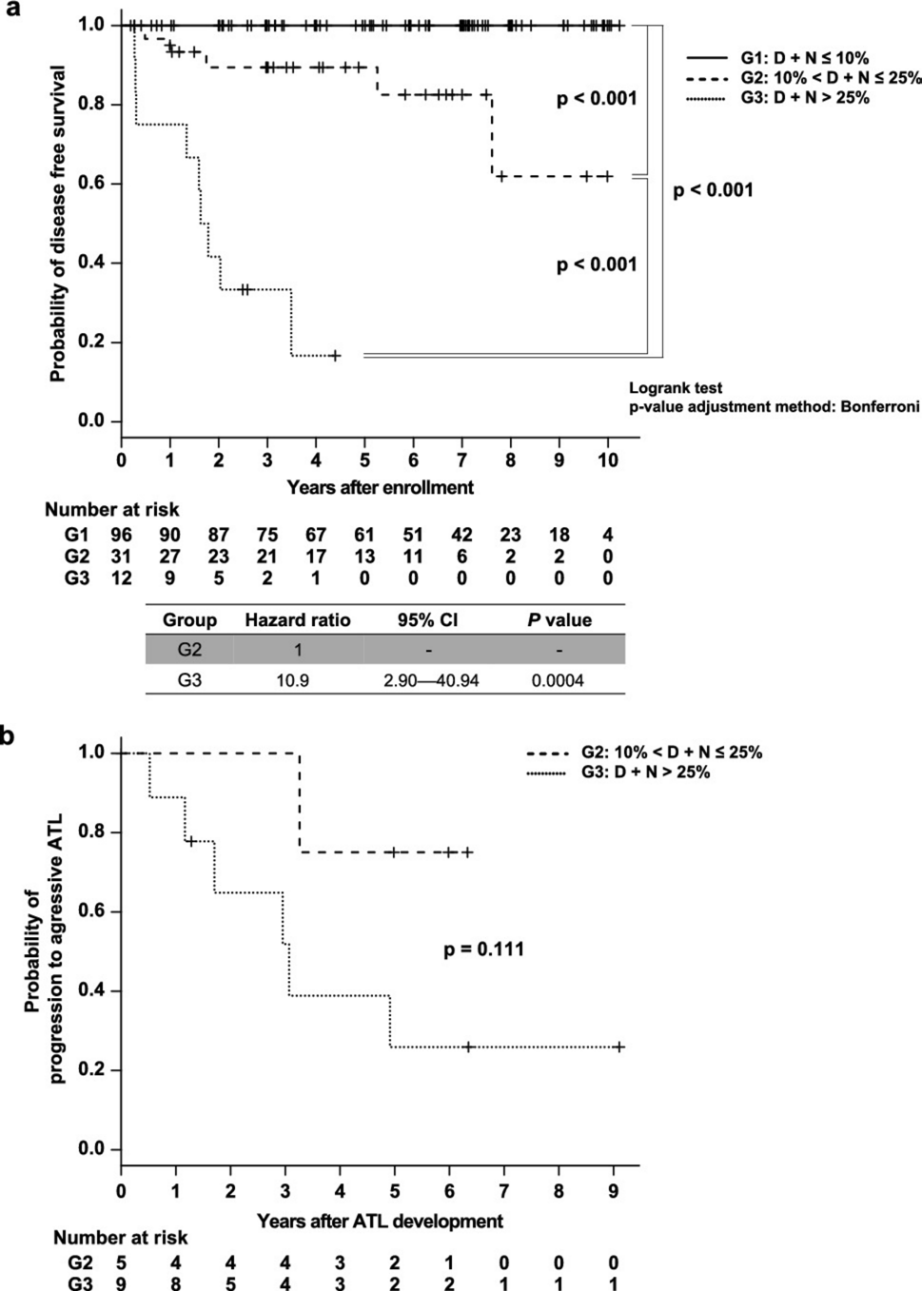
Probability of ATL disease-free or aggressive ATL-free survival for the three groups classified by the D + N percentage in HAS-Flow analysis Adult T-cell leukemia/lymphoma (ATL)-free and aggressive ATL-free survival analysis of the three groups classified based on HAS-Flow analysis was performed using the Kaplan–Meier method. (**a**) The number of years between the initial HAS-Flow analysis and the last peripheral blood smear was evaluated, and ATL development was defined as having ≥ 5% abnormal lymphocytes on examination. (**b**) Time from ATL development to aggressive ATL was assessed. The censored values (+) indicate the last known follow-up time of the participants who were alive without developing or aggressive ATL. A log-rank test with Bonferroni correction was performed, and the respective *p*-values are shown. The Cox proportional hazards model was used, and the hazard ratios and 95% confidence intervals are shown below the figure.

In addition, in the Results section, under the subheading ‘Relationships between the classification based on the D + N percentage of HAS-Flow analysis and ATL development or progression’,

“The hazard ratio calculated by the Cox proportional hazard model for G3 to G2 was 10.9 (95% CI: 2.90–40.94).”

now reads:

“The hazard ratio calculated by the Cox proportional hazard model for G3 to G2 was 12.7 (95% CI: 3.36–48.29).”

The original Article has been corrected.

