# Supplementary material for: A comprehensive assessment using multiple factors based on HAS-Flow analysis predicts ATL development and progression
Source: Sci Rep. 2025 Jul 22;15:26618. doi: 10.1038/s41598-025-10822-4 (PMC12284177; doi:10.1038/s41598-025-10822-4)
Supplement: Supplementary file 1 — Supplementary Information. [file 41598_2025_10822_MOESM1_ESM.pdf]

Supplementary material

Supplementary Table S1: Details of the HTLV-1 integration analysis

Supplementary Table S2: Details of primers used in PVL analysis

Supplementary Table S3: List of primers used in RAISING analysis

Supplementary Figure S1: Sequential changes of clinical markers in the case of G2

Supplementary Table S1. Details of the HTLV-1 integration analysis

HAS-Flow Group	Sample	HAS-Flow D+N (%)	PVL (%)	Chromosome	Position GRCh37	Orientation	Gene	Analysisg sequence
G2	A267	16.8	6.1	Chr6	88115808	5'-3'	1,883bp 5' upstream from CFAP206	TGAATGTAGCAAAAAACCCAAATAATAGTAGCTTAATAAAATAGGAGTTT
	A34	11.9	5.2	UD	UD	UD	UD	AATTTCGAGTTTAATATTTCTGTTGTTGATTTTGTTGTTTAGGAAGCTAC
	A47	11.5	4.0	UD	UD	UD	UD	CATTTTCATTTCACTTTGTCTTTAACTTTTTTGTTTGTTTCTGCTGTTTT
	A151	23.5	14.6	UD	UD	UD	UD	CGTAATAACTTGGATTGTCCCCCTTTCTGCTGGATTTTGTTTTTTTTGTT
	A114	16.6	7.3	Chr5	26600676	5'-3'	139,187bp 5' upstream from CCNB3P1	CTTGATAGTCTGTTATTAGGTACATATATGTTTATCACAATTATATCTTA
	A263	24.9	16.7	Chr10	90727485	3'-5'	Intron 1 of ACTA2	AATGGTCTTTACCAGACATGGCTTACAGTAACAAAATCATGAAACAAAAT
	A38	16.3	2.8	UD	UD	UD	UD	CATTGGTACTTTGCTTTCTTTGTATTTGTGTTCTGCTTATTTTACCTAGT
	A137	17.6	8.0	UD	UD	UD	UD	ACTAACAATTTAGCAAAATTAATAAAAAAATTTTAACGGTTCGCCTTTTC
	A296	20.0	10.5	UD	UD	UD	UD	TCTTTCGATCTTAATTCCTTTTTTTTTTTTTTTTGTTTGGTTTTTCGTGT
G3	A9	31.0	4.1	chr2	216704401	5'-3'	long intergenic non-protein coding RNA 607	ACGAGAGAGATGTATTTTGAGCCCCAGGGTGCTGGGATGAAGTTCTTTAA
	A25	31.5	NT	chr12	25692432	3'-5'	Intron 4 of LMNTD1	ATGCTGGATATTTGTTGTTGCTGCTCAGCATAAATATATTCTAAAGACTG
	A53	34.6	16.3	chr14	77930284	5'-3'	Intron 4 of AHSA1	ACCATGCATTCATAGGTGCTGGGTAGATTACCCACTCTTGAAGTCGTCAA
	A4	27.7	7.6	Chr19	51900658	5'-3'	NIFK pseudogene 6	AGAAAAGACAAGAGTGCATAAGATGGGTCCAGGGGACCAATGCTAGTATG
	A20	65.2	NT	chr20	38027850	5'-3'	49,816bp 5' upstream from RN7SL680P	AGTTCTCTCTTTAACTTGACCCCCATTGGGGCTGCCCCCCCATATCAGAA
	A210	75.5	63.7	chr20<br>chr5	26286761<br>46278040	5'-3'<br>3'-5'	96,892bp 5' upstream from MIR663AHG<br>581,558bp 5' upstream from HCN1	RWYCYTKYTSWSWDWBHDGKKBHKRRRYABKKWHHBKR
	A326	33.7	14.2	chr2	107640413	5'-3'	82,276bp 5' upstream of PPP1R2P5	TTTCTCGACCTCACTTTGGGGACTACACTGTGTCACTAATTGCCCTCTAG
	A87	32.6	NT	UD	UD	UD	UD	CTTTTCCACTTCCTTTTTGTTTTTTTTTTTTTCTTTTTTTTTTATTTTTT
	A320	31.7	15.1	UD	UD	UD	UD	GGTTCCCATTTTTTTTTTGTTTTTTTTTTTTTTTTAAAGGTGTGTTCTTT

Abbreviations:
UD, Unidentified; NT, Not tested.

Supplementary Table S2. Details of primers used in PVL analysis

HTLV-I pX region	Forward	5′-CGGATACCCAGTCTACGTGTT-3′
	Reverse	5′-CAGTAGGGCGTGACGATGTA-3′
	Probe	(FAM)–labeled pX2 probe 5′-CTGTGTACAAGGCGACTGGTGCC-3′
RNase P	The primers and the probe for the gene encoding RNase P were purchased from Applied Biosystems (#4316844)	

Supplementary Table S3. List of primers used in RAISING analysis

Name	Primer sequence (5'-3')
HTLV-1-F1	CAAGGCCTACCACCCCTCAT
HTLV-1-F2	CCTGACCCTGCTTGCTCAAC
HTLV-1-F3	AAAGTTCCACCCCTTTCCCTTT
Oligo-dT (23) Adaptor	ACAGCAGGTCAGTCAAGCAGTAGCAGCAGT TCGATAACATTTTTTTTTTTTTTTTTTTTTTTVN
ADP2	AGCAGTAGCAGCAGTTCGATAA

Supplementary Figure S1

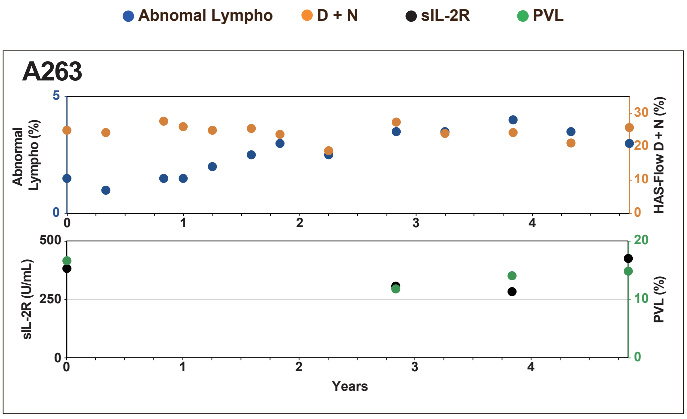

Figure S1: Sequential changes of clinical markers in the case of G2

Changes in four clinical markers (percentage of abnormal lymphocytes, D + N percentage of HAS-Flow, Soluble interleukin-2 receptors: sIL-2R, and Proviral load: PVL) in two cases in Group 2 are shown.
